# Efficacy of mailed surveillance invitations and telephone patient navigation to improve hepatocellular carcinoma surveillance uptake: study protocol of VIGILANT—a single-centre, two-arm randomised controlled trial

**DOI:** 10.1136/bmjopen-2024-097162

**Published:** 2025-06-30

**Authors:** Madhumitha Pandiaraja, Maria Qurashi, Hooshang Izadi, Maria Martinez, Rohini Sharma

**Affiliations:** 1 Imperial College Healthcare NHS Trust, Hammersmith Hospital, National Institute for Health Research Wellcome Trust Imperial Clinical Research Facility, London, UK; 2 Imperial College London, London, UK; 3 Department of Mechanical Engineering and Mathematical Sciences, Oxford Brookes University, Oxford, UK

**Keywords:** Patient Navigation, Hepatobiliary tumours, PUBLIC HEALTH, Health economics

## Abstract

**ABSTRACT:**

**Introduction:**

Hepatocellular carcinoma (HCC) is a leading cause of cancer-related mortality, and mortality rates have continued to rise despite advancements in treatment. Six-monthly ultrasound surveillance is recommended by professional bodies for early detection of HCC in high-risk cohorts. However, surveillance rates remain poor; only 20% of patients attend for regular surveillance. Population health outreach strategies may be able to enhance surveillance rates by addressing patient-related barriers to engagement with healthcare. Using a co-design approach, we have developed outreach material in tandem with patients, with the aim of boosting HCC surveillance. VIGILANT aims to compare the effectiveness of bespoke mailed surveillance invitations with or without patient navigators (PNs) in improving attendance rates at surveillance appointments.

**Methods and analysis:**

This is a two-arm randomised controlled trial that will recruit 652 participants. Participants will be patients with chronic liver disease or cirrhosis, who are eligible for HCC surveillance as defined by criteria from the National Institute for Health and Care Excellence and European Association for the Study of the Liver. Participants will be randomised (in a 1:1 ratio) to receive either (a) a mailed surveillance invitation or (b) a mailed invitation plus telephone call reminder from a PN 1 week prior to the appointment. The primary objective is to evaluate the impact of PNs and mailed invitations on attendance rates. Secondary objectives include rates of diagnosis of early-stage HCC among patients undergoing surveillance and cost-effectiveness of each arm.

**Ethics and dissemination:**

Approval has been sought to conduct the research from Aberdeen Research Ethics Committee (REC Reference: 335338). The study is sponsored by Imperial College London and funded by RM Partners (West London Cancer Alliance). Study findings will be presented at medical conferences and published in peer-reviewed journals.

**Trial registration number:**

NCT06635694.

**STRENGTHS AND LIMITATIONS OF THIS STUDY**

- Pragmatic randomised controlled trial design enabling evaluation of the effectiveness of studied interventions in a real-world setting.
- This trial adopted a co-design process in designing the mailed surveillance invitations disseminated to patients to place patients’ needs at the crux of the proposed interventions—a novel approach not previously used in hepatocellular carcinoma (HCC) surveillance studies.
- Study targets an ethnically diverse patient population, increasing applicability to regions across the UK.
- The use of outreach media may exclude marginalised and deprived populations that are typically hard-to-reach and disproportionately represented in the HCC surveillance target population.

## Introduction

 Hepatocellular carcinoma (HCC) is the third leading cause of cancer-related death globally.^1^ Mortality rates in the UK have tripled in the last 50 years and are projected to increase by 10% in the forthcoming two decades.^2^ Despite advancements in treatment, prognosis remains poor, with 5 year survival rates below 15%.^3^ One of the main drivers of poor prognosis is the detection of HCC at an advanced stage, precluding patients from curative therapy; over 75% of patients do not receive treatment with curative intent due to late stage diagnosis.^4^

Cirrhosis is a major risk factor for the development of HCC and is present in up to 90% of patients.^5^ Current guidelines from the European Association for the Study of the Liver (EASL), American Association for the Study of Liver Diseases and National Institute for Health and Care Excellence (NICE) recommend liver surveillance in patients with chronic liver disease with 6 monthly abdominal ultrasound scans to improve rates of early diagnosis and, consequently, improve survival outcomes.^6 7^ Despite recommendations, HCC surveillance is underused worldwide; in Europe, less than 50% of eligible patients receive surveillance.^8^ A recent meta-analysis in 2022 consisting of 29 studies yielded a pooled estimate of only 24% of patients with cirrhosis undergoing surveillance.^8^

Factors contributing to low surveillance uptake are manifold and may be broadly classified into patient-related, provider-related and system-level aspects.^8 9^ Several patient-related factors have also been identified, such as challenges with test scheduling, difficulty travelling to appointments and lack of understanding of the need for surveillance.^10 11^ Our work suggests that lack of knowledge and fear act as key barriers to HCC surveillance attendance.^12^ Understanding the reasons for surveillance failure allows for the development of more targeted interventions.

In the UK, current provision of HCC surveillance is ad hoc—eligible patients are identified and booked for a 6 monthly ultrasound scan by their designated clinician (usually a gastroenterologist, hepatologist or hepatology clinical nurse specialist). Patients receive an ultrasound appointment in the post, with no formal invitation or information about the surveillance test. We adopted a co-design process using workshops involving patient advocates with lived experience of liver disease and liver health charity representatives to identify barriers to surveillance attendance. Co-design is a collaborative approach, often employed in health service research, which actively involves stakeholders and service users to develop more effective solutions to a pre-specified problem.^13^ Reported barriers and patient preferences were subsequently used to develop user-friendly invitations and informational material.^12^ Focus groups also discussed telephone patient navigators (PN) as an additional intervention that may increase surveillance uptake.

Freeman *et al* describe eight key principles of PN, which highlight crucial considerations in designing the PN service, such as outlining a clear job scope for the navigator, ensuring cost-effectiveness and employing an individual with skills commensurate with the demands of the role.^14^ In line with these principles, PN is best delivered through establishment of a one-to-one relationship between a patient and navigator; the navigator being a trained staff member who can serve as a longer-term contact person working with the patient to identify and overcome hurdles in their individualised circumstances to access healthcare.

PN has been widely described as an effective intervention in enhancing participation rates for a wide range of screening programmes including breast, cervical, HCC and colorectal cancer, though these studies have been predominantly based in the USA.^15^ Two recent large-scale randomised controlled trials (RCTs) by Singal and colleagues have shown that mailed outreach and PN significantly improve HCC screening rates. Yet, in both studies, overall uptake remained poor, with less than 30% of at-risk patients receiving recommended biannual surveillance scans.^16 17^ A key limitation of these studies was the lack of co-design in developing mailed outreach material, which may render the intervention less relevant to the target population.

There has been no research to date on interventions developed through co-design aimed at improving HCC surveillance rates. The VIGILANT study aims to address this issue.

## Methods and analysis

### Study design

**Figure 1:**
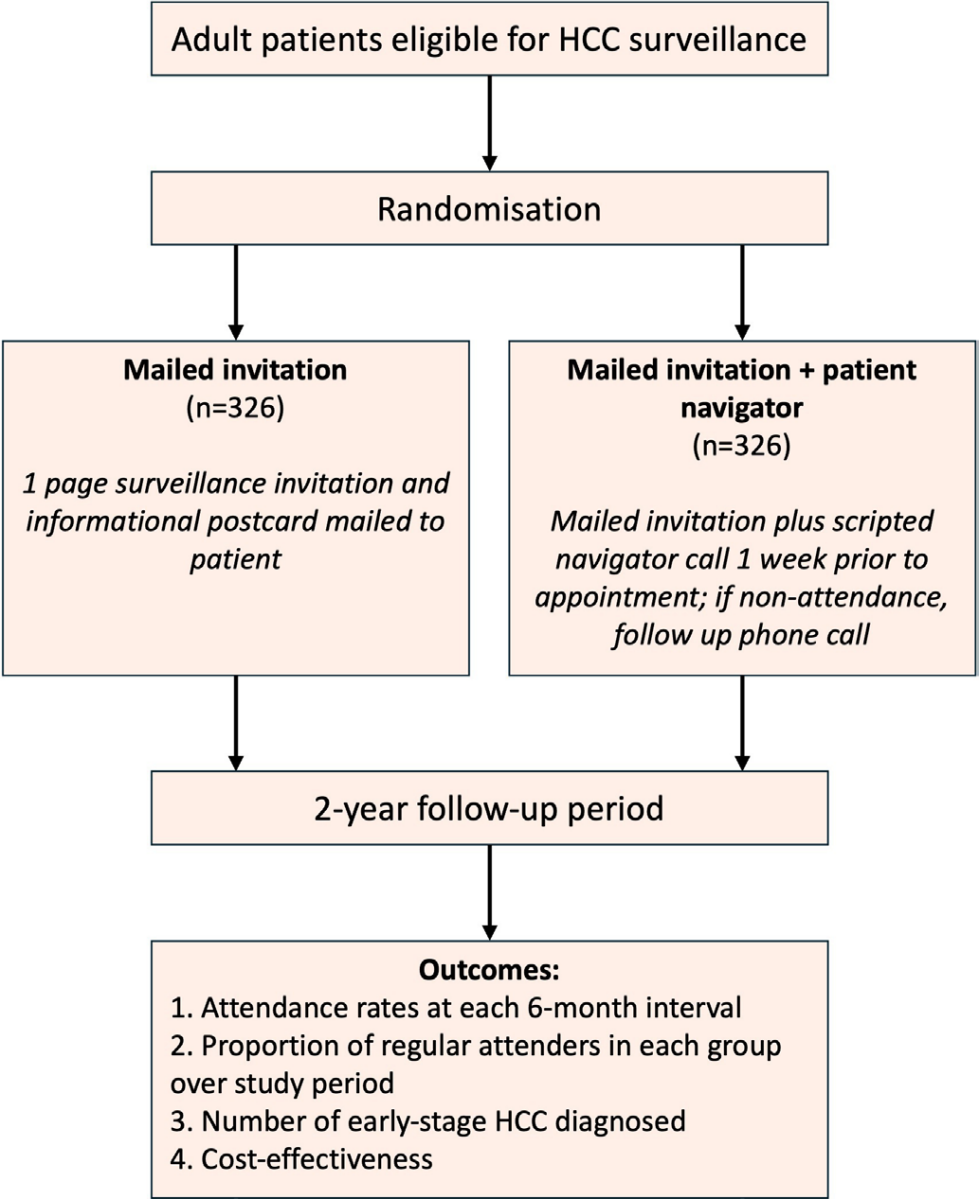
Flowchart of overall study timeline, allocation to trial arms and outcome measures. HCC, hepatocellular carcinoma.

VIGILANT is a RCT with two parallel groups, aiming to investigate the effect of mailed invitations with or without PN to improve the rate of HCC surveillance uptake over 2 years. Adults aged ≥18 years, at risk of developing HCC and suitable for HCC surveillance will be eligible to participate in the study. Surveillance uptake will be quantified based on patients’ attendance at their surveillance imaging appointments. In standard clinical practice in the UK, patients receive a random appointment time and date through the post, with subsequent provisions to reschedule if they are unable to attend the initial proposed appointment. To investigate the effect of enhanced outreach, trial participants will be randomised to one of the two following arms using a computer-generated number (figure 1):

1. Bespoke surveillance invitation, consisting of a one-page mailed invitation and an informational postcard.
2. Bespoke surveillance invitation plus PN, which comprises a scripted PN telephone call 1 week prior to surveillance appointment (in the event of non-attendance, a further follow-up PN call).

### Development of intervention measures

The mailed outreach material used in both trial arms, along with the patient navigation strategies, was iteratively designed in collaboration with patients, advocacy groups and behavioural specialists through workshops. Using the COM-B (Capability, Opportunity, Motivation) model of behaviour, we systematically mapped experiences shared by workshop participants to each component of the framework to identify key barriers that would need to be addressed through the study interventions. This facilitated the development of the letter invitation for HCC surveillance and the creation of a supplementary informational postcard accompanying the letter. Insight into the barriers faced by patients to participating in surveillance also shaped the content and structure of the script for telephone navigation.^12^

### Trial objectives

#### Primary objective

The primary objective is to evaluate the effect of bespoke mailed invitations and the addition of PN on the rate of patient attendance at surveillance appointments at each 6 monthly interval over a 2 year period.

#### Secondary objectives

Rate of diagnosis of early-stage HCC in each trial arm will be assessed and compared. A cost-effectiveness analysis will be undertaken to determine whether the introduction of PN in addition to bespoke mailed invitations to boost uptake of HCC surveillance is economically viable.

### Eligibility criteria

Participants will be recruited from a local patient database held by the liver care team at Imperial College Healthcare NHS Trust. A generic screening process will be undertaken through electronic chart review where the following data items will be collected: patient age, patient sex, home address postcode, whether the patient is a native English speaker and aetiology of liver disease. The database will only be accessed by the direct care team to identify potential trial participants.

Subjects will need to be deemed eligible for HCC surveillance as defined by NICE and EASL guidelines. This includes adults with liver cirrhosis classified as Child–Pugh A or B, and cirrhotic patients with Child–Pugh C disease awaiting liver transplantation.^18^ Comprehensive inclusion and exclusion criteria are outlined in table 1.^7^ The study aims to recruit 652 eligible participants.

**Table 1:** Inclusion and exclusion criteria

Inclusion criteria	Exclusion criteria
Age ≥18 years	Known previous or current diagnosis of HCC
Good or moderate performance status (ECOG, category 0–2)	Patients with Child–Pugh stage C cirrhosis who are not a candidate for liver transplantation
Child–Pugh stage A or stage B cirrhosis	Previous liver transplant
Child–Pugh stage C cirrhosis, awaiting liver transplantation	Score of >7 on the Clinical Frailty Scale
Non-cirrhotic chronic hepatitis B patients at intermediate or high risk of HCC (according to PAGE-B classes for Caucasian subjects, respectively 10–17 and ≥18 score points)	

ECOG, Eastern Co-operative Oncology Group; HCC, hepatocellular carcinoma.

The study will run between 2 January 2025 and 2 July 2027, and participant recruitment commenced on 1 February 2025.

### Study procedures

Study participants will be randomised into one of the two trial arms:

1. Mailed invitations only.
2. PN in addition to mailed invitations.

Randomisation will be carried out centrally using a computer-generated sequence. Concealed allocation will be used to reduce the risk of selection bias. Due to the nature of the interventions, no blinding will be employed.

The current surveillance recommended by NHS England comprises regular 6 monthly liver ultrasound scans for eligible patients.^7^ In routine clinical practice, the surveillance ultrasound scan will be requested by the subject’s responsible clinician. Ensuing ultrasound appointments will be flagged and booked by the clinical team using a bespoke call-and-recall system (RedCap).

#### Mailed invitations

Participants will be sent a formal invitation letter, endorsed by the patients’ general practitioner (GP), and an informational card via post to their home address (see online supplemental appendix 1). The mailed information leaflet will provide written and illustrative information on the ultrasound screening process and address frequently asked questions, including duration of the scan and level of discomfort. Two weeks prior to the ultrasound appointment date, patients will receive a reminder either via text message or letter. Following the scan, results will be automatically communicated to the patients’ hepatology care team and GP. Patients will also receive a copy of the results through mail or the ‘Patients Know Best’ app and be invited for a repeat ultrasound scan in 6 months if the scan is normal. In the event of an abnormal ultrasound scan, the patient will be contacted by their direct care team to organise further investigations.

**Figure 2:**
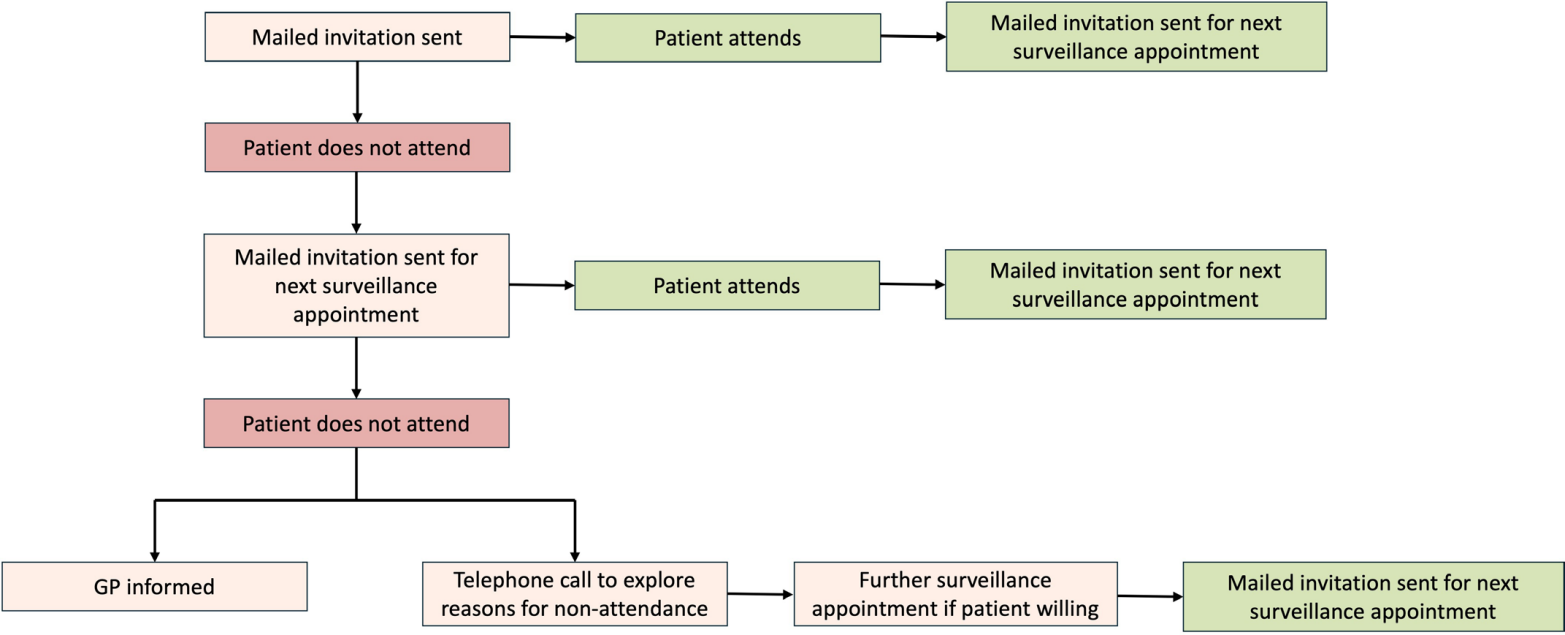
Flowchart of study procedures for participants allocated to trial arm receiving mailed invitations only. GP, general practitioner.

Non-attendance will be followed by a further mailed invitation for their next surveillance appointment. In the event of failure to attend two appointments, the patients’ GP will be informed, and the patient will be contacted by their direct care team to explore reasons for non-attendance. The study process for participants in this trial arm is summarised in figure 2.

#### Patient navigation

**Figure 3:**
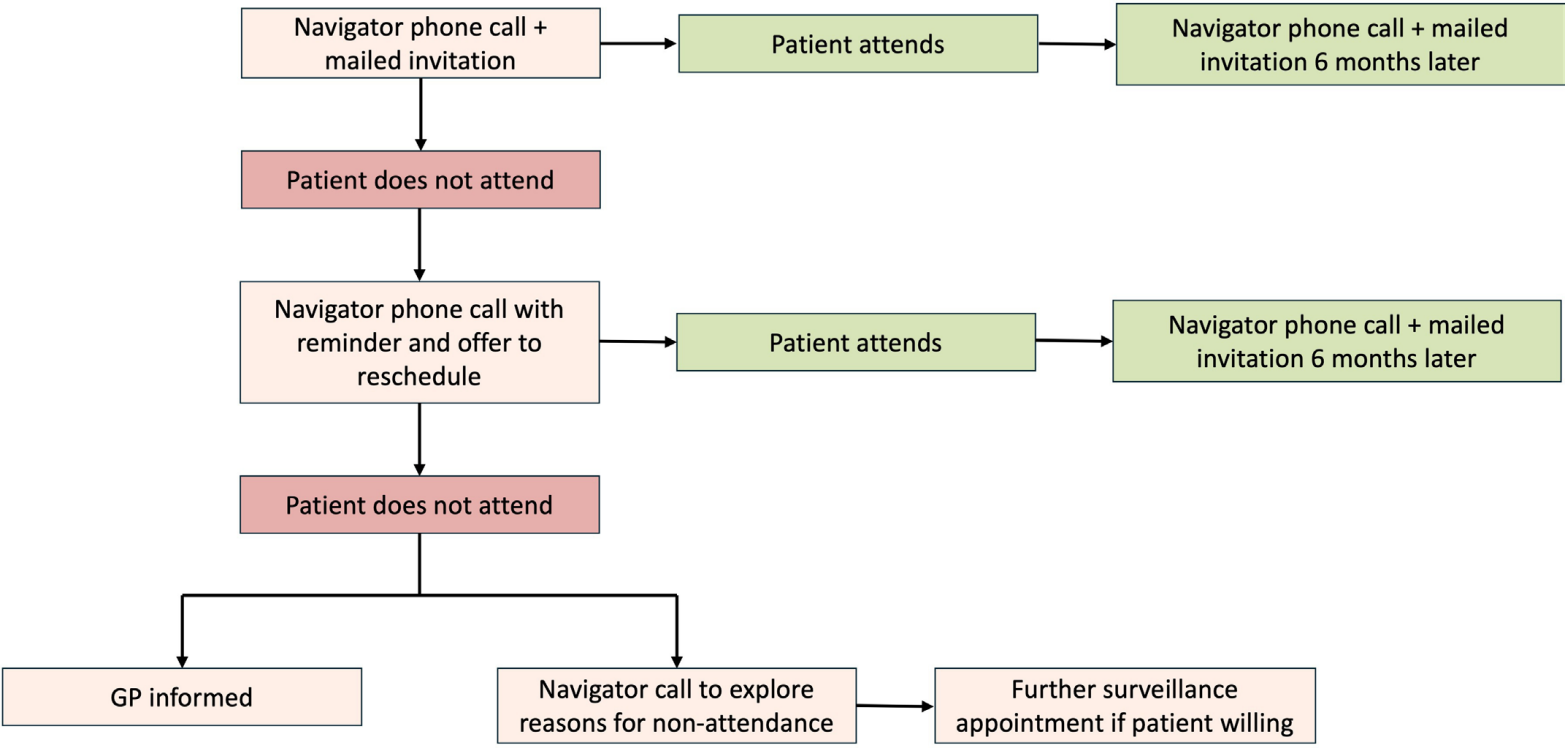
Flowchart of study procedures for participants allocated to trial arm receiving patient navigation. GP, general practitioner.

PNs are staff members who are already part of the patient’s existing direct care team (eg, usually a Band 4 administrator). For the purposes of this study, a specific staff member will be recruited to fulfil this role. Patients randomised to this trial arm will receive a mailed invitation plus a scripted telephone call from a PN (see online supplemental appendix 2). Patients will receive a telephone call from a PN 1 week prior to their surveillance appointment to (i) remind the patient of the appointment, (ii) offer to reschedule the appointment if the patient is unable to attend and (iii) address any concerns about the appointment. Additionally, PNs will call patients who have failed to attend a surveillance appointment to explore any barriers to attendance and offer solutions as to how these might be addressed. In the event of failure to attend two appointments, the patients’ GP will be informed, and the PN will call the patient to explore and record reasons for non-attendance. The study process for participants in the PN trial arm is summarised in figure 3.

### Outcomes

The primary outcomes of the study are (i) attendance rates at each 6 month interval and (ii) proportion of patients in each trial arm attending HCC surveillance regularly. Frequency of attendance at surveillance scans will be categorised into three groups: regular, infrequent and non-attendance.

- Regular attendance is defined as fulfilment of four or more HCC surveillance episodes over the study period, with no more than 8 months interval between surveillance appointments.
- Infrequent attendance is defined as attendance at some HCC surveillance appointments, but less than four episodes, or with intervals greater than 8 months between surveillance appointments.
- Non-attendance is defined as no attendance at any HCC surveillance appointments over the study period.

Secondary outcome measures are (i) proportion of patients in each arm diagnosed with early-stage HCC compared with late-stage HCC and (ii) comparative cost of interventions.

### Data collection

Data collected during the study will be recorded on REDCap, a secure web-based application for databases. Attendance at surveillance scans and HCC outcomes will be entered onto the database. At the end of the study (2 years), the following data will be extracted for analysis: age, sex, ethnicity, native language, Index of Multiple Deprivation quintile (based on patient’s home postcode), aetiology of liver disease, surveillance episodes, diagnosis of HCC and reasons for removal from surveillance pathway, if any.

### Statistical analysis

Descriptive statistics will be used to summarise baseline patient characteristics, primary and secondary outcomes from the two study groups. Continuous variables will be reported using number of observations, arithmetic mean, SD, median and minimum and maximum values; categorical variables will be summarised by frequencies and percentages. A two-way repeated measures analysis of variance will be performed, with time as a repeated factor, to compare the two intervention arms with respect to the primary outcome. Intention-to-screen analyses will be undertaken. Additionally, cost-effectiveness analysis will be performed using the return on investment (ROI) tool developed by Public Health England. The interactive ROI Tool can help estimate ROI, cost savings and health outcomes from specific public health interventions to guide authorities and policymakers to invest in initiatives that maximise health and economic benefits.^19^

### Sample size and power calculation

The target sample size is 326 participants in each trial arm. With a sample size of 652 participants in total, at an alpha of 0.05, the study has 85% power to detect a 10% absolute difference in surveillance rates between the intervention and control groups. This assumes a baseline surveillance completion rate of 10% in standard care, derived from a similar RCT in the USA comparing the effect of mailed outreach and PN on HCC surveillance participation.^16^

### Patient and public involvement

Key stakeholders, including patient advocacy groups (The British Liver Trust, The Hepatitis C Trust and Groundswell) and patient advocates, were consulted and engaged during the design of the protocol. To design the interventions in line with patients’ needs and preferences, we organised five co-production workshops (three face-to-face and two online) comprising patients with lived experience of liver disease and charity representatives. These workshops highlighted motivational factors and barriers to attending HCC surveillance among patients. Patient preferences and advice were sought to guide the development of the mailed letter and explanatory postcard about the surveillance process, including language used, informational content and addition of illustrations, to maximise the effectiveness of the outreach material.^12^

## Ethics and dissemination

Ethical approval to conduct the trial has been obtained from the Aberdeen Research Ethics Committee (Ref: 335338). A waiver of consent has been granted by the ethics board. The waiver of consent was deemed ethical as (i) the study carries minimal risk since all patients will receive HCC surveillance as per standard of care, (ii) the waiver of consent will not adversely affect the rights or welfare of the participants and (iii) necessitating consent would introduce volunteer bias, undermining the generalisability of the study and its validity as a population health study. Data will be stored in a secure environment with password protection, and every effort will be made to maintain confidentiality and anonymisation in line with General Data Protection Regulation. Findings of this trial will be shared internationally through publication in peer-reviewed journals and presentation at medical conferences.

## Supplementary material

10.1136/bmjopen-2024-097162 online supplemental file 1
