# Supplementary material for: Efficacy of mailed surveillance invitations and telephone patient navigation to improve hepatocellular carcinoma surveillance uptake: study protocol of VIGILANT—a single-centre, two-arm randomised controlled trial
Source: BMJ Open. 2025 Jun 30;15(6):e097162. doi: 10.1136/bmjopen-2024-097162 (PMC12211838; doi:10.1136/bmjopen-2024-097162)
Supplement: online supplemental file 1 [file bmjopen-15-6-s001.docx]

**Appendix 1: Copy of mailed invitation letter and informational postcard that will be disseminated to participants in both trial arms**

**Appendix 2: Patient navigator phone call script**

Example of phone call script (week prior to scan)
Hello my name is XXX I’m calling from XXX hospital, liver department.

I’m calling about your ultrasound scan which is booked for [date] [time] at [hospital]. I wanted to confirm you’ll be able to make it? Do you know where to go for the scan? Do you have any questions about the scan?

*If patient unavailable, offer alternate time/date*

Thank you. Bye

Example of phone call script (if patient did not attend scan)
Hello my name is XXX I’m calling from XXX hospital, liver department.

I’m calling about your ultrasound scan which was booked for [date] [time] at [hospital]. You didn’t attend so I’m calling to check if you wanted to reschedule.

Do you have any questions or concerns about the scan? Is there anything we can help with to get you to the scan?

*Offer alternate time/date*

Thank you. Bye

Example of phone call script – to be done by research team (if patient has two consecutive non-attendances)

Hello my name is XXX I’m calling from XXX hospital, liver department.

I’m calling about your ultrasound scan which was booked for [date] [time] at [hospital]. Our record shows you haven’t attended the last two scans.

The team are interested in understanding why some patients haven’t been able to make it in for the scan. Can I just check that you’ve received the appointment letters? And are you able to get to the hospital alright? How has your experience been with the department? Would you be interested in attending the scan going forward?

*If yes, is there any way we might be able to help you attend the scan? Offer alternate scan time/date*

*If no: is there a reason you don’t want to attend the scan going forward? Is there anything we could do differently?*

Thank you. Bye
