# Supplementary material for: Efficacy of mailed surveillance invitations and telephone patient navigation to improve hepatocellular carcinoma surveillance uptake: study protocol of VIGILANT—a single-centre, two-arm randomised controlled trial
Source: BMJ Open. 2025 Jun 30;15(6):e097162. doi: 10.1136/bmjopen-2024-097162 (PMC12211838; doi:10.1136/bmjopen-2024-097162)
Supplement: Supplementary file 2 [file bmjopen-2024-097162.reviewer_comments.pdf]

PEER REVIEW HISTORY

BMJ Open publishes all reviews undertaken for accepted manuscripts. Reviewers are asked to complete a checklist review form and are provided with free text boxes to elaborate on their assessment. These free text comments are reproduced below.

ARTICLE DETAILS

Title (Provisional)

Study Protocol of VIGILANT: a single-centre, two-arm randomised controlled trial evaluating the efficacy of mailed surveillance invitations and telephone patient navigation to improve hepatocellular carcinoma surveillance uptake

Authors

Pandiaraja, Madhumitha; Qurashi, Maria; Izadi, Hooshang; Martinez, Maria; Sharma, Rohini

---

VERSION 1 - REVIEW

**Reviewer**	**1**
**Name**	**Beal, Eliza W**
**Affiliation**	**The Ohio State University**
**Date**	**29-Dec-2024**
**COI**	**None**

The authors propose a timely and relevant study, "Study Protocol of VIGILANT: a single-centre, two-arm randomised controlled trial evaluating the efficacy of mailed surveillance invitations and telephone patient navigation to improve hepatocellular carcinoma surveillance uptake." I have some minor comments below that may help the authors clarify the proposal.

The authors define the arms as follows: "participants will receive either (Figure 1): 1. Bespoke surveillance invitation, consisting of a 1-page mailed invitation and an informational postcard 2. Bespoke surveillance invitation plus PN, which comprises a scripted PN telephone call 1 week prior to surveillance appointment (in the event of non-attendance, a further follow-up PN call)." Does this mean that included patients are only those who are following with a provider and for whom surveillance appointments have already been made? In that case, does the appointment consist of the ultrasound appointment and a meeting with a physician at the same time? Or two appointments? Were the patients consulted on the proposed date and time of the scheduled appointments? In this case the patients are already VERY involved, and this is a significant limitation of this study. Either way the proposed process should be more clearly described.

How will patients be identified and enrolled for this study? This process should be clearly described.

The length of the study period should be more clearly described. Under data collection it suggests that the study period is two years, however this doesn't make sense for the outcomes as they are currently defined. For example, the authors stated that, "Surveillance scans will be categorised into 3 groups: regular, infrequent and non-attendance. Regular attendance is defined as fulfilment of 4 or more HCC surveillance episodes over the study period, with no more than 8 months interval between surveillance appointments." Based on that definition a patient could be defined as regular attendance if they had four scans, each nearly 8 months apart, which would be a total of 31 months (longer than 2 years).

In the statistical analysis section the authors mention that, "cost-effectiveness analysis will be performed using the return on investment tool developed by Public Health England." Could this tool be described or another reference cited?

The authors could consider including some additional references.

Reviewer 2

Name Brain, Kate

Affiliation Cardiff University, Cochrane Institute of Primary Care and Public Health

Date 28-Feb-2025

COI None

Overall, this is a well-written protocol describing a novel trial of a behavioural intervention to improve liver surveillance adherence versus an optimised standard of care control. However, several methodological details are lacking:

Introduction - please expand what is meant by 'co-design' and with whom this was carried out.

Study Design - please provide study duration dates. Randomisation procedures are lacking in detail and should be under a separate subheading, detailing whether concealed allocation and any blinding will be used.

Eligibility Criteria - there should be an additional subheading explaining participant consent procedures, including withdrawal.

Intervention - please provide further details of the behavioural intervention, including how the scripted telephone call was developed, whether it was based on behavioural theory, and whether training was developed for the patient navigator delivering the intervention.

Outcomes - no details provided on economic evaluation measures or their analysis.

Sample Size - this should be detailed under a separate subheading, including the basis for assumptions such as 10% difference in surveillance uptake.

Ethics - Please provide the trial registry number.

Can the authors also please consider including additional sections describing the assessment of adverse events, data management/monitoring and governance, and a brief discussion.

VERSION 1 - AUTHOR RESPONSE

Reviewer: 1
Dr. Eliza W Beal, The Ohio State University

Comments to the Author:
The authors propose a timely and relevant study, "Study Protocol of VIGILANT: a single-centre, two-arm randomised controlled trial evaluating the efficacy of mailed surveillance invitations and telephone patient navigation to improve hepatocellular carcinoma surveillance uptake." I have some minor comments below that may help the authors clarify the proposal.

The authors define the arms as follows: "participants will receive either (Figure 1): 1. Bespoke surveillance invitation, consisting of a 1-page mailed invitation and an informational postcard 2. Bespoke surveillance invitation plus PN, which comprises a scripted PN telephone call 1 week prior to surveillance appointment (in the event of non-attendance, a further follow-up PN call)." Does this mean that included patients are only those who are following with a provider and for whom surveillance appointments have already been made? In that case, does the appointment consist of the ultrasound appointment and a meeting with a physician at the same time? Or two appointments? Were the patients consulted on the proposed date and time of the scheduled appointments? In this case the patients are already VERY involved, and this is a significant limitation of this study. Either way the proposed process should be more clearly described.

Response:
Thank you for highlighting the ambiguity that this section raises.

To clarify, participants included in the study will be patients who are already receiving care under the specialist Hepatology service at any one of the constituent hospitals under Imperial College Healthcare NHS Trust and meet the eligibility criteria for HCC Surveillance according to NICE and EASL guidelines. Surveillance appointments are scheduled as part of their routine care and not created purely for the purposes of the study.

The appointment referenced in the intervention consists of only the ultrasound appointment, and patients receive separate appointments with their physician (which may be at more frequent, or less frequent intervals than their imaging appointments depending on clinical indication). In standard practice, patients are not consulted on the date and time of their proposed appointment but are able to re-schedule if they are unable to attend. The interventions proposed in the study (i.e. mailed informational postcard, patient navigation) are layered on top of this routine scheduling process.

We have revised the manuscript to incorporate these clarifications (see Methods and Analysis section, page 5, lines 164-172).

How will patients be identified and enrolled for this study? This process should be clearly described.

Response:
Eligible patients will be identified from a local database held by the liver care team at Imperial College Healthcare NHS Trust. This local database is held by the care team for the purposes of routine care administration and will only be accessed by the Direct Care Team to identify participants who qualify for HCC surveillance. We have added text to describe this (see Methods and Analysis section, page 6, lines 206-210).

The length of the study period should be more clearly described. Under data collection it suggests that the study period is two years, however this doesn't make sense for the outcomes as they are currently defined. For example, the authors stated that, "Surveillance scans will be categorised into 3 groups: regular, infrequent and non-attendance. Regular attendance is defined as fulfilment of 4 or more HCC surveillance episodes over the study period, with no more than 8 months interval between surveillance appointments." Based on that definition a patient could be defined as regular attendance if they had four scans, each nearly 8 months apart, which would be a total of 31 months (longer than 2 years).

Response:
The study period remains fixed at 2 years. For example, if the patient was only attending scans at 8 monthly intervals, then within the study period they would only have 3 scans or less so the patient would be classified as having infrequent attendance.

In the statistical analysis section the authors mention that, "cost-effectiveness analysis will be performed using the return on investment tool developed by Public Health England." Could this tool be described or another reference cited?

Response:
We have briefly described the tool and cited a reference to the Tool User Guide which describes the tool's purpose, and how it is used in the context of a different public health intervention to inform the commissioning of sexual and reproductive services in the UK (see Statistical Analysis section, page 8, line 289-292).

The authors could consider including some additional references.

Response:
We have included pertinent additional references related to the development of the behavioural interventions employed in the trial, previous studies that have informed our sample size calculations, and sources explaining the intended economic analyses that will be undertaken as part of the study.

Reviewer: 2
Dr. Kate Brain, Cardiff University
Comments to the Author:
Overall, this is a well-written protocol describing a novel trial of a behavioural intervention to

improve liver surveillance adherence versus an optimised standard of care control. However, several methodological details are lacking:

Introduction - please expand what is meant by 'co-design' and with whom this was carried out.

Response:
We agree that it would be helpful to define 'co-design' for readers and have added the following (Page 4, Line 130-134). The preceding sentence has been modified to reflect more clearly who the co-design workshops were carried out with – patients with liver disease and liver health charity representatives. The following text has been added:

"Co-design is a collaborative approach, often employed in health service research, that actively involves stakeholders and service users to develop more effective solutions to a pre-specified problem."

Study Design - please provide study duration dates. Randomisation procedures are lacking in detail and should be under a separate subheading, detailing whether concealed allocation and any blinding will be used.

Response:
The study duration dates have been added to the Methods section (page 6, lines 216-217). Randomisation procedures have been described in greater detail under the 'Study Procedures' sub-heading (page 7, lines 226-228).

Eligibility Criteria - there should be an additional subheading explaining participant consent procedures, including withdrawal.

Response:
The waiver of consent obtained for this study has been detailed in the sub-section on 'Ethics and Dissemination'. Withdrawal procedures thus do not apply here.

Intervention - please provide further details of the behavioural intervention, including how the scripted telephone call was developed, whether it was based on behavioural theory, and whether training was developed for the patient navigator delivering the intervention.

Response:
We have expanded on how the interventions were designed and developed through co-design and using the COM-B model. This can be found under the sub-heading 'Development of Intervention Measures' (page 5-6, lines 179-195).

Outcomes - no details provided on economic evaluation measures or their analysis.

Response:
We have briefly described the economic evaluation tool that will be used for analysis and cited a reference to the Tool User Guide which describes the tool's purpose, and how it is used in the context of a different public health intervention to inform the commissioning of sexual and reproductive services in the UK (see Statistical Analysis section, page 8, line 289-292).

Sample Size - this should be detailed under a separate subheading, including the basis for assumptions such as 10% difference in surveillance uptake.

Response:

We have added the following text under a new sub-heading.

"The target sample size is 326 participants in each trial arm. With a sample size of 652 participants in total, at an alpha of 0.05, the study has 85% power to detect a 10% absolute difference in surveillance rates between the intervention and control group. This assumes a baseline surveillance completion rate of 10% in standard care, derived from a similar RCT in the USA comparing the effect of mailed outreach and patient navigation on HCC surveillance participation." (Page 8, lines 293-298).

Ethics - Please provide the trial registry number.

Response:
The trial registry number for the study is NCT06635694 (Page 2, Line 56).

Can the authors also please consider including additional sections describing the assessment of adverse events, data management/monitoring and governance, and a brief discussion.

Response:
We believe that, as this is a behavioural intervention study, managing adverse events is not applicable to the trial. We have added further detail on information governance under the 'Ethics and Dissemination' section (Page 9). We hope that through the appraisal of existing literature within the Introduction section, and description of how the behavioural interventions were developed within the Methods section, we have provided sufficient context for readers to understand the rationale for and implications of our study.
